# Anemia in a middle aged female with aortitis: a case report

**DOI:** 10.1186/s13104-015-1572-3

**Published:** 2015-10-22

**Authors:** Shabneez Hussain, Salman Naseem Adil, Shahid Ahmed Sami

**Affiliations:** Section of Haematology, Department of Pathology and Microbiology, The Aga Khan University Hospital, Stadium Road, P.O. Box 3500, Karachi, 74800 Pakistan; Department of Surgery, The Aga Khan University Hospital, Karachi, Pakistan; Fatimid Foundation, 393, Britto Road, Garden east, Karachi, 74800 Pakistan

**Keywords:** Idiopathic aortitis, Refractory anemia, Granulomatous aortitis, Valvular heart disease

## Abstract

**Background:**

Idiopathic aortitis is among the most common causes of non-infectious aortitis, which rarely presents with anemia.

**Case presentation:**

Here we report a case of a 49-year-old muhajir female who presented with shortness of breath and easy fatigability for the past 6 months. Physical examination revealed pallor and a diastolic murmur in the aortic region. Echocardiography showed thickened and calcified aortic and mitral valves, severe aortic regurgitation and dilatation of ascending aorta. She was advised aortic valve replacement and was referred to a haematologist due to concomitant anemia. Complete blood counts revealed haemoglobin: 7.7 gm/dl, mean corpuscular volume (MCV): 78 fl, mean corpuscular haemoglobin (MCH):23 pg, total white cell count: 9.0 × 10^9^/L and platelet count: 227 × 10^9^/L. Erythrocyte sedimentation rate (ESR) was 100 mm/hr. There was suspicion of myelodysplastic syndrome, but could not be confirmed as the patient refused bone marrow and cytogenetic studies. She was given erythropoietin, folic acid and ferrous sulphate. Following relatively prolonged therapy, her haemoglobin level increased to approximately 9.0 gm/dL. She was transfused with packed red cells and underwent aortic valve and ascending aorta replacement. The ascending aorta was dilated and aortic wall markedly thick and irregular. Histopathology of the resected aorta revealed granulomatous aortitis. She was prescribed prednisolone, which resulted in further incremental rise of haemoglobin to 13.1 gm/dL. One month later, she developed complaints of blurred vision in the right eye and was diagnosed with central retinal vein occlusion. She was treated with antiplatelet agents and her vision improved. After 3 months, she was asymptomatic and her haemoglobin level rose to 11.2 gm/dL without hematinic therapy or blood transfusion. She was begun on anticoagulant therapy and remains clinically stable.

**Conclusion:**

We report a case of idiopathic aortitis with presumed diagnosis of anemia of chronic disease exhibiting a transient response towards steroid therapy post-valvuloplasty.

## Background

Aortitis, characterized by inflammation of aortic wall [1], may be secondary to infectious (tuberculosis and syphilis) and non-infectious diseases (Takayasu’s arteritis [2], giant cell arteritis [3] and rarely Behcet’s syndrome [4], sarcoidosis [5], rheumatoid arthritis [6], systemic lupus erythematosus [7]). Conditions that predispose to aortitis include connective tissue diseases and diabetes mellitus [8]. The prevalence of biopsy proven aortitis is reported to be 6.1 % with approximately 73 % cases being idiopathic and 14 % cases due to giant cell arteritis (GCA) [9]. Another study has shown that idiopathic aortitis and GCA are the most common causes of aortitis, especially affecting women in their 7th decade of life [10]. A recent study revealed that 31 % of non-infectious aortitis are GCA and 59 % are isolated [11]. In another study, 47 % patients with isolated GCA in ascending aorta experienced distal aortic events during an average follow up of 8 years [12].

Aortitis predominantly affects women with a mean age of 63–72 years [13, 14]. Sending aortic biopsy specimens for histopathology is not practiced in certain institutions [8]. This hinders the assessment of prognosis in patients with aortitis, which is worse than that of an ordinary aortic aneurysm, resulting in a higher risk of postoperative complications [14, 15].

Idiopathic aortitis, also known as “nonspecific aortitis,” “unclassified aortitis,” and “aortitis of obscure etiology”, [16] is a frequently diagnosed entity which has a prevalence of 4.3–8.4 % [9, 13, 14] and it is predominantly seen in women with age ranging from 32 to 83 years [13]. It specifically affects ascending thoracic aorta and is incidentally diagnosed on histopathology obtained from procedures undertaken due to aneurysm or aortic dissection [1]. However, a criterion for diagnosing idiopathic aortitis does not exist [8, 9]. The histological features of idiopathic aortitis are similar to GCA and Takayasu’s arteritis (TA) [9]. Miller et al. have reported that giant cells are seen in all cases of GCA and TA, while granulomas were present in 21 % cases of GCA and all cases of TA [10]. In idiopathic aortitis, 76 % cases showed giant cells and 19 % cases had granulomas [10]. It is associated with systemic diseases in 31 % cases [13]. Idiopathic aortitis is an under diagnosed entity since it is usually asymptomatic [10] and mainly diagnosed on the basis of lack of extra aortic arteritis and autoimmune connective tissue disorders from other causes of aortitis [10].

Studies have reported the association of mild normocytic and normochromic anemia with GCA in one-third to one half cases [17–19]. Hall et al. have reported the association of mild anemia with TA [20]. Literature review revealed that anemia is rarely seen in patients with idiopathic aortitis. We report a case of idiopathic aortitis whose initial presentation was anemia.

## Case presentation

A 49-year-old muhajir female presented to the cardiology outpatient clinic of an urban tertiary hospital with a 6 month history of shortness of breath and easy fatigability. There was no history of fever, weight loss or night sweats. Physical examination revealed extreme pallor and a diastolic murmur in the aortic region. There was no visceromegaly. Family history was unremarkable. There was no history of past interventions. She was afebrile and her blood pressure was 118/70 mmHg. She had received one unit of packed red cells. Transthoracic echocardiography revealed severely dilated left atrium and mildly dilated right atrium. Ejection fraction was approximately 50 % with mild global hypokinesia. Aortic valve was thickened, calcified and sclerotic with absence of stenosis and severe (eccentric) aortic regurgitation was seen. Mitral valve was also thickened, calcified with absence of stenosis and severe (eccentric) mitral regurgitation was identified. Tricuspid and pulmonary valves were normal with no stenosis. The ascending aorta was dilated. There were no thrombi, vegetations, pericardial effusion or intracardiac shunts. Chest X-ray revealed pulmonary edema and she was given furosemide. Six months later, her dyspnea worsened and she was advised valvuloplasty. However, due to anemia, her surgery was postponed and she was referred to a haematologist.

Complete blood counts revealed haemoglobin: 7.7 gm/dL, haematocrit: 26 %, MCV: 78 fl, MCH: 23 pg, total white cell count: 9.0 × 10^9^/L, absolute neutrophil count: 6.8 × 10^9^/L and platelet count: 227 × 10^9^/L. Peripheral smear showed hypochromic red cells and right-shifted neutrophils. Reticulocyte count was 4.2 %. There was absence of red cell fragmentation and features of hemolysis on peripheral smear. ESR was 100 mm/hr. Direct antiglobulin test was negative. Anti-nuclear, anti-mitochondrial and anti-smooth muscle antibodies were also negative. Serum ferritin levels were 387 ng/mL, serum vitamin B 12 levels were 765 pg/mL and red cell folate levels were 1545 ng/mL. A suspicion of myelodysplastic syndrome was raised and she was advised bone marrow biopsy and cytogenetics, which was not done due to patient’s refusal. She was prescribed erythropoietin, folic acid and ferrous sulphate for 12 weeks after which haemoglobin level rose to 9.3 gm/dL.

**Fig. 1:**
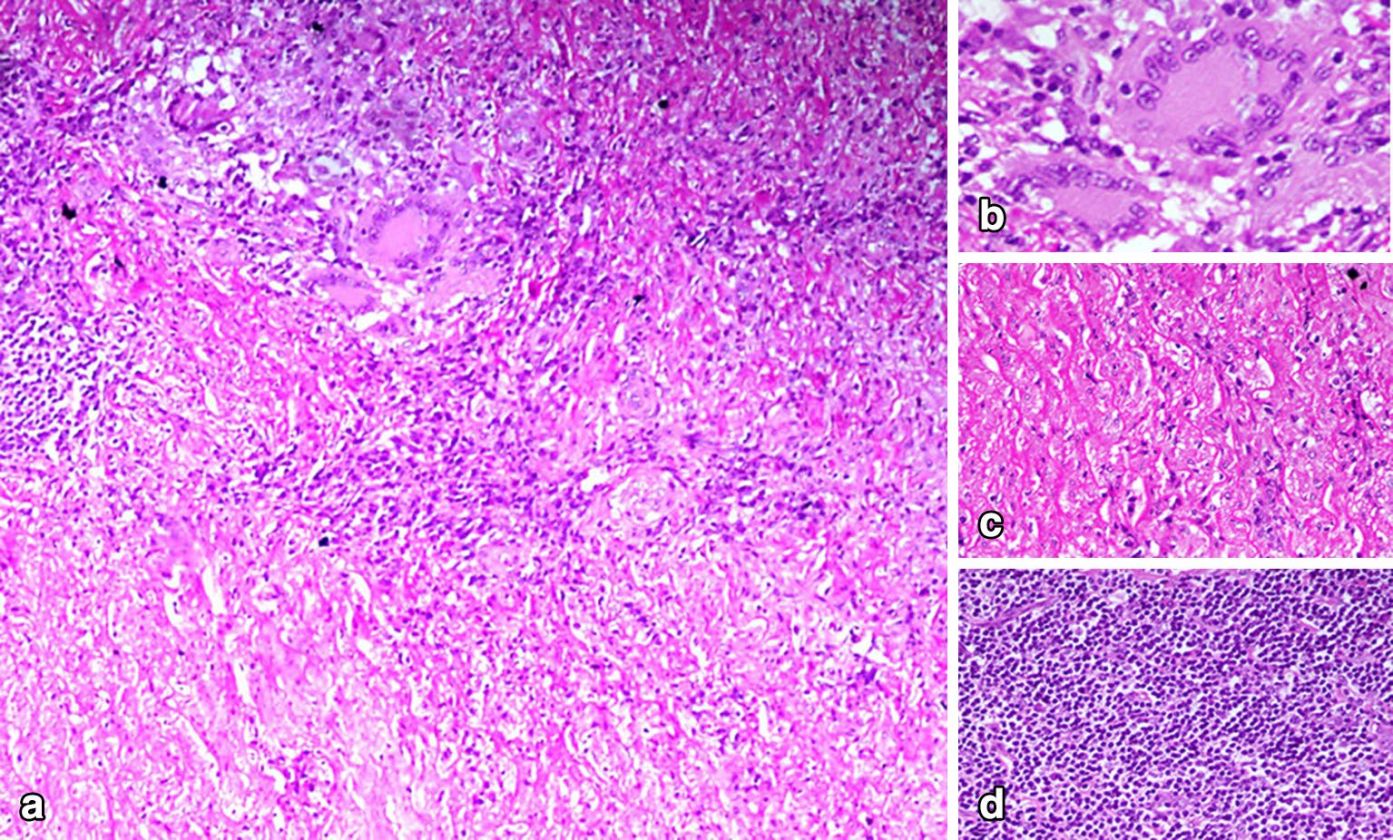
**a** Low power (20×) view of thickened and fibrosed aortic wall showing chronic granulomatous inflammation. **b** High power (100×) view of a granuloma composed of epitheloid histiocytes, surrounding lymphocytes, plasma cells and Langhan’s type giant cells. **c** High power (100×) view of fibrosed wall showing wavy collagen bundles. **d** High power (100×) view of lymphoid aggregates

She developed chest pain radiating to the mandible due to which she was prescribed glyceryl trinitrate and was advised urgent surgical intervention. She was transfused with packed red cells prior to surgery and her post transfusion haemoglobin was 11.3 gm/dl. She underwent aortic valve replacement, ascending aorta replacement and mitral valve repair. The ascending aorta was grossly abnormal with dilatation and adhesion to pericardium and surrounding structures. The wall of ascending aorta was markedly thick and irregular. The ascending aorta resection and valve cusps were sent for histopathology. It revealed full thickness vessel wall showing moderate degree of fibrous intimal thickening (Fig. 1). The medial and adventitial layers showed scattered aggregates of lymphocytes along with granuloma formation. These granulomas were composed of histiocytes with few interspersed multinucleated giant cells. There was absence of caseation necrosis. Ziehl–Neelsen stain for acid fast bacilli and periodic acid-Schiff for fungi was applied and found to be negative. The overall features were suggestive of granulomatous aortitis.

Serum acetylcholinesterase levels were 57 IU/L (normal range up to 52 IU/L). Mantoux test was negative. Venereal disease and rapid plasma reagin tests were also negative. The level of anti-cyclic citrullinated peptide was less than 7 μ/mL (normal range up to 17 μ/mL). Postoperatively, on the basis of histological findings she was started on prednisolone (1 mg/kg/day) for 6 weeks and her haemoglobin improved from 11.6 to 13.1 gm/dL. Azathioprine (150 mg daily) was also begun concomitantly with prednisolone as a long term immunosuppressive therapy.

One month later, she developed complaints of blurred vision in the right eye. She was referred to an ophthalmologist and was diagnosed with central retinal vein occlusion. Clopidogrel (75 mg daily) was added to aspirin (75 mg daily), which she was receiving from time of surgery and her vision improved. Currently, she is receiving warfarin along with aspirin and remains clinically stable.

## Discussion

The cause of aortitis in our case appears to be idiopathic since our patient did not fulfill the clinical criteria of GCA and TA has been regarded as unlikely. Presence of anemia, visual manifestations, marked elevation of erythrocyte sedimentation rate changes, female gender, age group (50–70 years) and involvement of aorta favors the diagnosis of GCA, while the absence of headache, involvement of temporal artery, jaw claudication, scalp tenderness and facial pain negates it [17]. TA, also known as pulseless disease (usually affecting upper limbs), is usually seen in young women before 30 years of age with heart failure, hypertension, arthralgia, palpitations, headache, carotid bruit, visual manifestations and involvement of aorta [17, 21, 22]. Granulomas comprising of Langhans-type giant cells are seen in both GCA and TA [17]. In the absence of fever, weight loss, night sweats and negative results of Mantoux test, tuberculosis was excluded as a cause of aortitis. Other causes of aortitis such as syphilis, RA and sarcoidosis have also been excluded. Therefore, this patient was diagnosed as idiopathic aortitis.

This case study signifies the importance of diagnosing underlying chronic disease in a patient with refractory anemia. The patient initially presented with haemoglobin of 7.7 gm/dL and hypochromic indices. She had limited response to hematinic therapy with improvement of haemoglobin levels to 9.3 gm/dL. She had to be transfused in order to undergo cardiothoracic surgery. Due to blood transfusion, serum iron levels and total iron binding capacity were not performed and the cause of anemia was presumed to be anemia of chronic disease. Also, poor nutritional status secondary to chronic heart disease and the picture of hypochromic microcytic anemia point to the possibility of concomitant iron deficiency as evidenced by the “normal” haemoglobin values obtained after transfusion. Indeed, the reticulocyte count responded to iron therapy.

There are hypotheses behind the association of anemia and idiopathic aortitis, such as myelodysplastic syndrome and autoimmune diseases [23, 24]. Vasculitis affecting small vessels [25] have been seen more frequently compared to those affecting large vessels [26]. In patients with cytopenias, particularly refractory anemia, myelodysplastic syndrome is a prime differential. Since the patient refused bone marrow evaluation, she was initially treated with erythropoietin therapy based on the assumption that she may have an underlying refractory anemia associated with myelodysplastic syndrome or anemia of chronic disease. Co-existence of more than one pathology is not uncommon. Steurer et al. [27] have reported two cases of myelodysplastic syndrome suffering from large vessel arteritis. Both patients showed a significant improvement in haematological parameters, although one of them transformed to acute myeloid leukemia [27].

The transient response of anemia to steroid therapy still remains unexplained since the hemoglobin level rose from 11.6 to 13.1 gm/dL, but returned to 11.2 gm/dL thereafter. The anemia was responding to nutritional replacement therapy prior to prednisolone, but the slow rise was most likely a result of poor cardiovascular function as expected in a patient with chronic disease. The improved hemodynamics and circulation after valvular replacement surgery may have contributed to the improved hemoglobin level.

Steroid therapy in this patient proved beneficial as idiopathic aortitis is known to have a good prognosis and though it is responsive to anti-inflammatory therapy, the prognosis was found to be good even in the absence of therapy [10]. It has been seen that 17 % of the patients with idiopathic aortitis go on to develop new aneurysm therefore follow up is essential in these cases [13]. Since idiopathic aortitis remains largely under diagnosed it is important to identify common clinical features in these patients. Miller et al. have reported that 33 % patients in their study have experienced back or chest pain while fever, fatigue and Raynaud’s phenomenon were rarely seen [10]. The most important finding was that 40 % of the patients with idiopathic aortitis were asymptomatic [10] with absence of underlying systemic diseases which may aid in diagnosis.

Understanding the pathophysiology of idiopathic aortitis may help in guiding therapy towards anemia associated with this disease. Proinflammatory cytokines are released by the activated CD4 helper T lymphocytes producing IL-2, interferon gamma growth mediators and metalloproteinase [26]. These induce damage resulting in medial necrosis inciting a secondary response in the form of histiocytic reaction seen as multinucleated giant cells [28] in histopathology specimens. The underlying immune mechanism may also explain the cause of anemia in these patients and their favorable response to immunosuppressive therapy.

Central retinal vein occlusion is known to be associated with systemic vasculitis [29, 30]. Of all the large vessel vasculitis, TA is known to cause central retinal vein occlusion [31] while GCA has been associated with retinal artery occlusion [32]. However, Chu et al. reported concurrent retinal artery and vein occlusion in a patient with GCA [33]. Literature review did not reveal any association of idiopathic aortitis with retinal vein or artery occlusion. Even though the patient in this case study did not fulfill the criteria of either TA or GCA, she did develop central retinal vein occlusion. This may have been due to cranial arteritis with secondary central vein thrombosis.

There are several limitations in this case report that need to be highlighted. Although anemia was the cause of presentation, the association between anemia and aortitis could not be described clearly. The patient did not undergo bone marrow aspiration so the etiology of anemia remained uncertain. Echocardiography, which revealed the valvular disease and ascending aortic aneurysms, was only an incidental finding that later lead to the diagnosis of aortitis. Also, when she presented a month later with blurred vision, she had another episode of inflammation. Perhaps at that time inflammatory markers should have been checked and the patient should have been seen by a rheumatologist. The association between anemia and aortitis is hypothetical as it could have been two separate pathologies occurring in this patient. It is possible that it was anemia of chronic disease resulting from chronic inflammatory arteritis, but this could not be proven.

## Conclusion

In conclusion, an evaluation directed towards an underlying pathology should be considered in any patient with refractory anemia and valvular heart disease as steroid therapy may have a beneficial effect on haematological parameters. Such patients should always be under regular surveillance since recurrence of aneurysms secondary to idiopathic aortitis in patients devoid of steroid therapy has been documented [13]. Further studies are required for evaluating the association between anemia and idiopathic aortitis.

## Consent

Since the patient was illiterate, informed verbal consent was obtained from her. Written informed consent was given by the patient’s son for publication of this Case Report and any accompanying images. A copy of the written consent is available for review by the Editor-in-Chief of this journal.

## Abbreviations

MCH: mean corpuscular haemoglobin
MCV: mean corpuscular volume
ESR: erythrocyte sedimentation rate
TA: Takayasu’s arteritis
GCA: giant cell arteritis
